# Recent Progress in Thermoplastic Polyurethane/MXene Nanocomposites: Preparation, Flame-Retardant Properties and Applications

**DOI:** 10.3390/molecules29163880

**Published:** 2024-08-16

**Authors:** Yao Yuan, Weiliang Lin, Lulu Xu, Wei Wang

**Affiliations:** 1 Fujian Provincial Key Laboratory of Functional Materials and Applications, School of Materials Science and Engineering, Xiamen University of Technology, Xiamen 361024, China; weilianglin318315@163.com; 2 School of Chemical Engineering, University of New South Wales, Sydney, NSW 2052, Australia; lulu.xu1@unsw.edu.au; 3 School of Mechanical and Manufacturing Engineering, University of New South Wales, Sydney, NSW 2052, Australia

**Keywords:** thermoplastic polyurethane, MXene, modification, flame retardants, mechanism

## Abstract

MXene, a promising two-dimensional nanomaterial, exhibits significant potential across various applications due to its multilayered structure, metal-like conductivity, solution processability, and surface functionalization capabilities. These remarkable properties facilitate the integration of MXenes and MXene-based materials into high-performance polymer composites. Regarding this, a comprehensive and well-structured up-to-date review is essential to provide an in-depth understanding of MXene/thermoplastic polyurethane nanocomposites. This review discusses various synthetic and modification methods of MXenes, current research progress and future potential on MXene/thermoplastic polyurethane nanocomposites, existing knowledge gaps, and further development. The main focus is on discussing strategies for modifying MXene-based compounds and their flame-retardant efficiency, with particular emphasis on understanding their mechanisms within the TPU matrix. Ultimately, this review addresses current challenges and suggests future directions for the practical utilization of these materials.

## 1. Introduction

The rapid development and widespread use of polymeric materials have raised concerns about their fire safety (flammability) issues. As a significant polymer, thermoplastic polyurethane (TPU) is ubiquitous in various fields, including transportation, coatings, automotive, and electronics, due to its high ductility, strong adhesiveness, good processability, and high compression strength [1,2]. TPU is a block copolymer consisting of alternating soft and rigid segments composed of carbon, hydrogen, and oxygen. These segments influence the ‘quality’ of phase separation between blocks, contributing to TPU’s outstanding mechanical properties [3,4]. Nevertheless, the presence of both hard and soft segments also makes TPU highly prone to combustion, posing significant fire safety risks in everyday applications. In particular, the release of toxic gases like carbon monoxide (CO) and hydrogen cyanide (HCN), along with soot particles, presents a significant fire hazard [1]. Therefore, understanding the flammability issues of TPU and developing effective flame-retardant strategies is crucial for expanding its safe use in high-risk applications.

**Figure 1:**
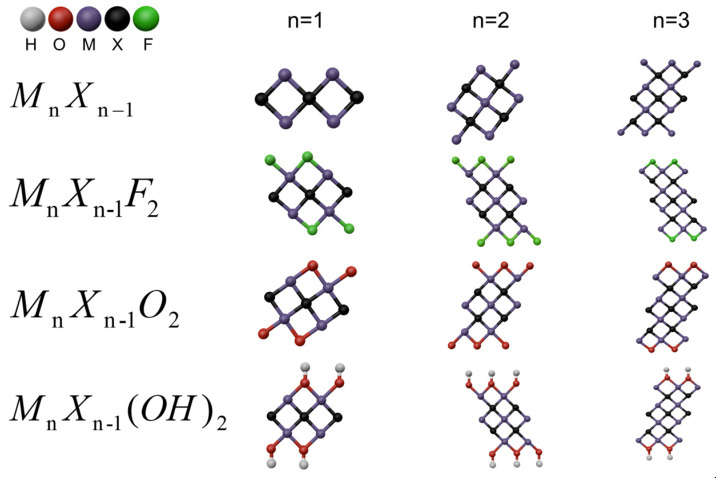
Models of bare MXenes (M_n_X_n-1_), fluorine-terminated MXenes (M_n_X_n-1_F_2_), oxygen-terminated MXenes (M_n_X_n-1_O_2_), and hydroxyl-terminated MXenes [M_n_X_n-1_(OH)_2_]; n varies from 2 to 4. Color code: blue = metal; deep gray = X; red = oxygen; gray = hydrogen; green = fluorine.

MXenes, a novel family of two-dimensional (2D) transition metal carbides, carbonitrides, and nitrides, have emerged as a groundbreaking class of materials since their discovery in 2011 [5,6,7]. Known for their distinctive layered structure and outstanding mechanical properties, MXenes exhibit remarkable properties that make them highly appealing for a wide range of applications in energy storage [8], electromagnetic interference (EMI) shielding [9,10], microwave absorption [11], and various other fields [12,13,14,15]. MXenes are characterized by the general formula M_n+1_X_n_T_x_, where M and X denote transition metal and carbon and/or nitrogen elements, respectively, along with surface terminations [16,17]. As illustrated in Figure 1, MXenes are primarily synthesized through the etching of A-group elements from MAX-phase metal ceramics using hydrogen fluoride (HF) and other etchants. MXenes are regarded as a promising group of functional nanofillers, valued for their impressive flexibility and layered structures that facilitate uniform dispersion within polymer materials. This facilitates the fabrication of numerous polymer nanocomposites that enhance mechanical properties [18,19], EMI shielding [20,21], and flame retardancy [22,23].

MXene has emerged as an ideal reinforcing filler for thermoplastic polyurethane (TPU) composites, offering advantages beyond those of graphene. MXene particles often achieve good crystallinity after high-temperature treatment while maintaining a layered structure with excellent structural stability [24]. Their large surface area and high aspect ratio are crucial for effective reinforcement in the TPU matrix. These properties enable strong interfacial interactions between MXene sheets and polyurethane chains, leading to improved load transfer and mechanical strength. Moreover, the early transition metal compounds found in MXene exhibit favorable catalytic effects, effectively hindering smoke release and promoting char formation during combustion. MXene also has rich terminal groups that are easier to modify and integrate into the TPU matrix [25,26,27,28]. As a result, MXene has attracted significant attention as a novel addition to the flame-retardant material arena. However, MXene nanosheets also face challenges such as easy aggregation, restacking, and oxidation in the air, which can hinder their application in certain fields [29,30]. To address these problems, the functional modification and/or structural design of MXene is necessary to enhance its dispersibility in the TPU matrix, leading to high-performance nanocomposites. Based on the analysis presented in this study, MXene-based nanofillers demonstrate significant potential and have garnered increased interest for use in fire-safe TPU composites.

Although recent advances in MXene for energy conversion, storage applications, photocatalytic applications, and supercapacitors have been extensively investigated, there have also been some preliminary studies on the use of MXene in flame-retardant polymer materials [31,32,33]. However, the improvement in TPU flame-retardant and smoke suppression properties using modified MXene has rarely been reported until now. In this review, we explore recent advancements in the methods for modifying MXenes, as well as the current state and future potential of MXene/thermoplastic polyurethane (TPU) nanocomposites. We delve into various modification techniques and synergistic approaches that enhance the flame-retardant properties of MXenes. This review examines MXene-based functionalized products as effective flame retardants, discussing their modification principles, fire safety performance, optimization strategies, and mechanisms of action. Additionally, this review provides an overview of the critical bottlenecks faced by MXene/TPU nanocomposites and suggests future directions for development, which are crucial for the progress of MXene-based nanomaterials.

## 2. Flame-Retardant Mechanisms for TPU

### 2.1. Synthesis and Combustion Characteristics of Polyurethane

**Figure 2:**
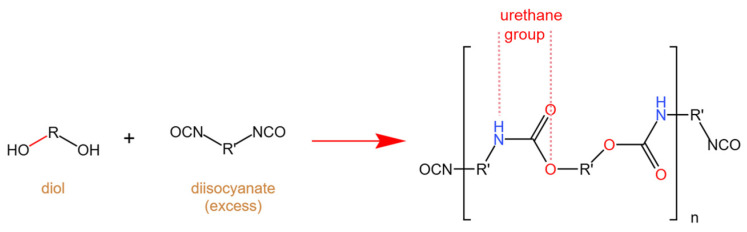
Basic reaction scheme for urethane formation.

**Figure 3:**
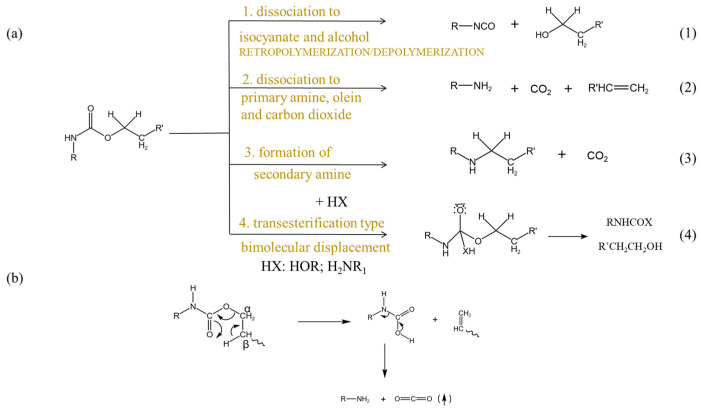
Thermal degradation mechanism of (**a**,**b**) the urethane segment.

Polyurethanes (PUs) are among the most versatile and widely utilized polymers in commercial applications. They are synthesized through a polyaddition reaction involving diisocyanates and polyols. This process primarily involves two key components: the isocyanate group (-NCO) from diisocyanates and the hydroxyl group (-OH) from polyols. The fundamental urethane formation reaction is illustrated in Figure 2. PU is a versatile polymer known for its exceptional mechanical properties, comprising soft and hard segments primarily made of carbon, hydrogen, and oxygen [34,35]. This composition makes TPU highly flammable, as these elements readily participate in combustion reactions [36]. A large number of researchers have reported that the more easily formed urethanes are, the more unstable they become, and the more prone they are to cleavage. Over the past 40 years, extensive research has been conducted to explain the thermal decomposition process of polyurethanes. Relevant work on commercial polyurethanes has been summarized by Ravey and Pearce [37]. Dyer et al. studied models of carbamates [38] and identified three pathways for the initial low-temperature (180–300 °C) cleavage of carbamates. Additionally, Saunders, Frisch, and Pielichowski [39] summarized four types of reactions occurring during the thermal degradation of polyurethanes (Figure 3). The material can ignite easily and sustain burning, posing a serious fire hazard. When heated, the interactions between molecular chains weaken, transforming the structure from a network to a linear one. As the temperature rises, the carbamate bonds decompose, forming short-chain and small molecules [40]. Continued heating causes the polyol soft segments to decompose, with the resulting small molecules diffusing into the flame zone and mixing with air to form a flammable mixture. Once a critical point is reached, gas-phase combustion occurs, and the feedback heat further accelerates the thermal decomposition, creating a vicious cycle. Upon exposure to heat, the interactions among molecular chains weaken, leading to a transition in the structure from a network to a linear configuration. Continued heating causes the decomposition of polyol soft segments, allowing the resulting small molecules to diffuse into the flame zone, where they mix with air, forming a combustible mixture. After combustion, TPU produces a minimal amount of char residue that is porous and cracked, and it lacks density, rendering it ineffective for insulation purposes. Throughout combustion, TPU undergoes substantial melt dripping, resulting in a significant decrease in melt viscosity that prevents it from sustaining its own weight. Simultaneously, the polyurethane material often generates smoke, carbon monoxide, carbon dioxide, and nitrogen-containing volatiles [41,42]. Effective flame retardants can substantially reduce the fire hazards of TPU by inhibiting the concentration of decomposition products during the initial combustion stage [43].

Rigid polyurethane foam (RPUF) and flexible polyurethane foam (FPUF) are widely utilized polymer materials renowned for their excellent insulation properties in various industrial and residential applications. Despite their advantages, both RPUF and FPUF possess an inherent flammability due to their polymeric composition, which facilitates rapid ignition and sustained combustion. RPUF, characterized by its low density and high porosity, ignites readily when exposed to high temperatures or open flames. The resulting combustion is marked by intense and rapid flame spread, driven by the high surface area of the material and the ease with which it generates fuel vapors. This is further exacerbated by the composition of foam, which supports vigorous burning and heightens the associated fire hazards. On the other hand, FPUF exhibits a different combustion profile due to its flexible nature and unique chemical formulation. While it also ignites relatively easily, the rate and intensity of flame spread can differ from RPUF, influenced by factors such as density and cell structure. Despite these differences, both types of polyurethane foam exhibit significant flammability. Therefore, ongoing advancements in material development and fire-resistant technologies are essential to improving fire safety in applications involving polyurethane foams.

### 2.2. Condensed-Phase Flame-Retardant Mechanism

The condensed-phase flame-retardant mechanism of TPU is a crucial approach to enhancing its flame resistance. This mechanism primarily involves actions occurring in the solid phase of polymer materials, focusing on delaying or inhibiting thermal decomposition, forming protective layers, and reducing heat transfer [44]. Here are the key aspects: (1) Stabilizers within the flame-retardant formulation help to maintain the integrity of the char layer, preventing it from cracking or disintegrating. A stable char layer reduces the release of volatile combustible gases, thereby slowing down the overall combustion process. (2) The char layer formed in the condensed phase acts as a thermal shield. It absorbs and dissipates heat, lowering the temperature of the material beneath it. This thermal barrier helps to protect the polymer matrix from further degradation and ignition. (3) Some flame retardants function as catalysts in the condensed phase, accelerating dehydration and cross-linking reactions that facilitate char formation. These catalytic effects enhance the efficiency of char formation, resulting in a more robust and effective barrier against combustion. (4) The labyrinth effect relies on creating a convoluted structure within the material, acting as a barrier to the spread of flames and the flow of heat and gases. This effect can be achieved by incorporating graphene-like nanomaterials or specific flame retardants and fillers that increase the material’s complexity at a microscopic level.

### 2.3. Gas-Phase Flame-Retardant Mechanism

The gas-phase flame-retardant mechanism is essential for enhancing the fire resistance of TPU composites [45,46]. This approach focuses on disrupting the combustion process in the gas phase, where the actual burning occurs. During combustion, flame retardants release substances that interfere with the radical chain reactions responsible for flame propagation. These substances target and neutralize highly reactive radicals such as hydrogen (H•), hydroxyl (OH•), and oxygen (O•) radicals, which are crucial for maintaining the combustion process [47]. Some flame retardants decompose when exposed to high temperatures, releasing inert gases such as water vapor, carbon dioxide, or nitrogen. These inert gases dilute the concentration of flammable gases in the combustion zone, effectively reducing the overall flammability of the material [46,48,49]. Moreover, certain flame retardants undergo an endothermic reaction during decomposition, absorbing heat from the surroundings. This heat absorption decreases the temperature within the combustion zone, thereby slowing the rates of pyrolysis and gas-phase reactions.

### 2.4. Synergistic Flame-Retardant Mechanism

Combining various flame retardants, such as phosphorus-based compounds, nitrogen-based additives, and synergists like antimony compounds or metal oxides, enhances the fire resistance of TPU [50,51]. Each component plays a role in different fire suppression aspects, including lowering heat release, reducing smoke production, and mitigating toxic gas emissions. The synergistic effect is pivotal as it amplifies the overall fire safety performance beyond what individual flame retardants can achieve independently. This approach typically improves adherence to fire safety standards and regulations.

## 3. Synthesis and Modification of MXenes

### 3.1. Synthesis of MXenes

MXenes are innovative nanomaterials obtained through the selective etching of MAX phases, which are layered ternary carbides, nitrides, or carbonitrides characterized by alternating layers of M, A, and X elements [14]. In the unit cell of MAX phases, the bonding between M and A is weaker compared to M-X bonds, thereby increasing the susceptibility of M-X bonds to disruption. Hence, the selective etching of the A-phase from MAX has emerged as the predominant method for producing MXenes.

#### 3.1.1. Fluoride-Containing Etching Methods

**Figure 4:**
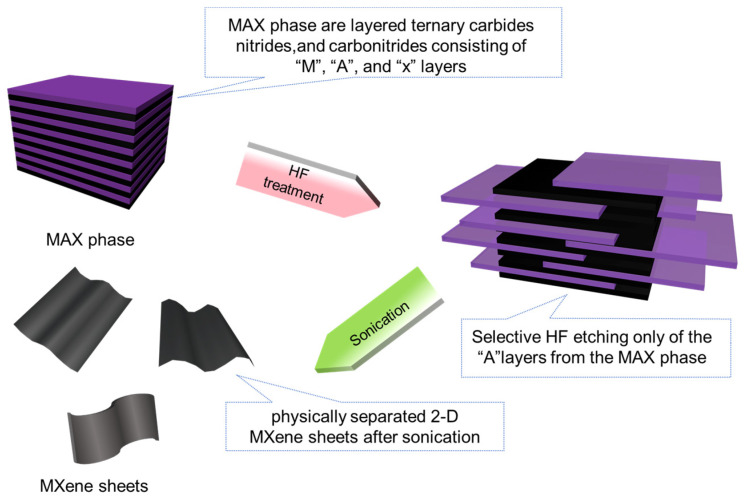
Schematic representation of HF acid etching process of MAX phase.

Fluoride-containing etching methods are fundamental to the synthesis of MXenes, a class of advanced nanomaterials derived from MAX phases. These methods primarily involve the use of hydrofluoric acid (HF), which selectively removes M layers (often aluminum) from MAX phases while leaving A and X layers intact [52]. HF etching operates through the formation of soluble fluoroaluminate complexes, ensuring precise control over the etching process. The conditions, including concentration, temperature, and duration, are meticulously regulated to achieve high selectivity and efficiency without compromising the structural integrity of the remaining layers. Due to the corrosive and toxic properties inherent in hydrofluoric acid, it is essential to implement stringent safety precautions and specialized handling procedures. These fluoride-containing etching techniques not only facilitate the production of MXenes with tailored properties such as high conductivity and mechanical strength but also support their applications in energy storage, catalysis, and composite materials for various technological advancements. Currently, HF etching remains the predominant method for synthesizing MXenes. Naguib et al. [53] successfully obtained Ti_2_C and Ti_3_CN by selectively etching aluminum from Ti_2_AlC and Ti_3_AlCN using hydrofluoric acid at concentrations of 10% and 30%, respectively, as illustrated in Figure 4. Yuan et al. [54] described a highly effective Ti_3_C_2_ MXene nanofiber (NF) catalyst produced through a hydrolysis process of bulk Ti_3_AlC_2_, followed by HF etching.

#### 3.1.2. Alkaline Etching Strategy

The Alkaline etching strategy for MXenes is a pivotal method in their synthesis, focusing on the selective removal of aluminum layers from MAX phases using alkaline solutions [55,56]. Typically, potassium hydroxide (KOH) is utilized due to its strong alkalinity and ability to dissolve aluminum layers while preserving the integrity of the remaining MAX-phase structure. The process starts with submerging MAX-phase materials like Ti_3_AlC_2_ in a concentrated KOH solution under controlled parameters. This treatment facilitates the exfoliation of layered structures, yielding MXene nanosheets. Subsequent steps involve thorough washing and purification to remove residual etchants and achieve purified MXene nanosheets suitable for further functionalization or composite material fabrication. Li et al. [57] employed a hydrothermal alkaline approach to selectively etch Ti_3_AlC_2_, with the goal of synthesizing NaOH-Ti_3_C_2_T_x_. Their research showed that aluminum was successfully extracted from Ti_3_AlC_2_ at 270 °C using a 27.5 M sodium hydroxide (NaOH) solution. As a result, the process yielded Ti_3_C_2_T_x_ nanosheets with surface terminations containing both hydroxyl and oxygen groups, achieving a purity of 92 wt%. The NaOH-Ti_3_C_2_T_x_ nanosheets demonstrated a non-uniform interlayer spacing of around 1.2 nm, contrasting with the 0.93 nm spacing observed in Ti_3_AlC_2_.

#### 3.1.3. Electrochemical Etching Strategy

The electrochemical etching strategy for MXenes involves using an electrochemical setup to selectively remove the A layer, typically aluminum, from MAX phases [58]. In this process, a MAX-phase material is used as the working electrode in an electrolyte solution. When voltage is applied, aluminum atoms are oxidized and dissolved into the electrolyte, leaving behind the M and X layers to form MXene. This method allows for precise control over etching by adjusting voltage, current density, and electrolyte composition. It produces MXenes with fewer defects and improved structural integrity compared to traditional chemical methods and is safer and more environmentally friendly by avoiding the use of highly corrosive acids. Yang et al. [59] described the electrochemical etching of Ti_3_AlC_2_ using an electrolyte composed of 1 M NH_4_Cl and 0.2 M tetramethylammonium hydroxide to produce single- or double-layer Ti_3_C_2_T_x_.

## 4. MXene-Based Flame-Retardant Polyurethane Systems

MXenes, discovered in 2011 by Yury’s group, represent a novel family of two-dimensional transition metal carbides, nitrides, and carbonitrides. These materials have garnered significant attention due to their exceptional properties, which include high electrical conductivity, a large surface area, excellent mechanical properties, and versatile surface chemistry. Table 1 offers a comprehensive collection of graphite oxide (GO), carbon nanotubes (CNTs), and MXene-based flame-retardant options for enhancing the mechanical properties of the TPU matrix. The results indicate that MXene-based flame retardants significantly enhance mechanical properties. These attributes make MXenes highly suitable as functional fillers for enhancing fire safety and EMI shielding in TPU composites, potentially broadening the application range of MXenes in TPU materials.

### 4.1. Preparation of Thermoplastic Polyurethane/MXene Nanocomposites

#### 4.1.1. Solution Casting

In the pursuit of advancing high-performance polymer nanocomposites, the solvent casting method has emerged as a prominent technique for fabricating thermoplastic polyurethane (TPU)/MXene nanocomposites. This approach leverages the unique properties of MXenes to enhance the functional characteristics of TPU, a versatile polymer renowned for its mechanical flexibility and durability. Once both TPU and MXene solutions are prepared, they are combined to ensure the uniform distribution of MXenes throughout the TPU solution. This blending process often involves methods such as stirring, ultrasonication, or high-shear mixing to achieve a well-dispersed MXene phase within the TPU matrix. The blend is subsequently poured into molds or onto substrates to form films or other intended shapes. For instance, Yu et al. [65] achieved a uniform blend by mixing MXene, modified with a cationic agent, with TPU in dimethylformamide (DMF). Subsequently, TPU/MXene composites were produced by adding the dispersion solution into water.

#### 4.1.2. Melt Blending

Melt blending is a prominent technique for fabricating thermoplastic polyurethane (TPU)/MXene nanocomposites, leveraging the inherent compatibility of polymers and fillers in the molten state. This method involves the incorporation of MXenes into TPU matrices under elevated temperatures to ensure thorough dispersion and integration. In this process, MXenes are initially introduced into the TPU matrix using high-shear melt blending techniques, typically performed in a twin-screw extruder or a high-capacity melt mixer. This approach offers several advantages, including scalability and the potential for improved interfacial interactions between the TPU and MXenes, ultimately leading to advanced performance characteristics in various applications.

### 4.2. Utilization of Pristine MXene

Pristine MXenes, as two-dimensional nanomaterials typically derived from MAX phases through selective etching processes, have demonstrated significant potential as flame-retardant additives in thermoplastic polyurethane (TPU) systems [25,27,64]. The surfaces of MXenes, which include functional groups such as O, OH, and F, provide a hydrophilic surface that facilitates their incorporation with TPU during the mixing process. Jiang et al. [66] successfully developed shape memory PU/MXene paper, composed of thermoplastic polyurethane and pristine MXene, through a facile electrospinning process. This material exhibits rapid self-extinguishing behavior without melt dripping. Additionally, PU/MXene paper provides a stable detection signal, has ideal early fire warning responses, and functions as a circuit fuse based on its shape recovery properties.

**Figure 5:**
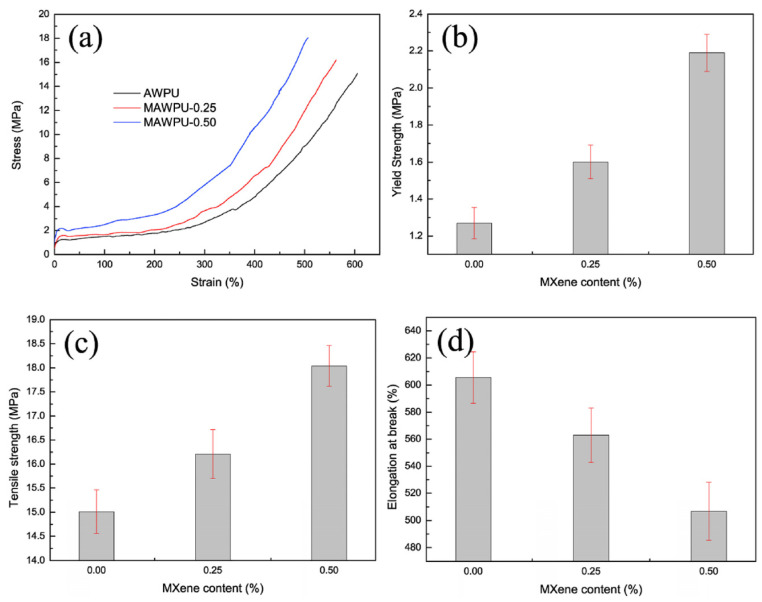
The tensile properties of the MXene/PU composites: (**a**) the typical stress–strain curves; (**b**) yield strength; (**c**) tensile strength; (**d**) elongation at break [68]. Copyright 2018. Reproduced with permission from Elsevier Science, Ltd.

In the preparation of high-performance composites, a large dosage of filler is often necessary to achieve the desired enhancements in properties such as thermal stability, conductivity, or flame retardancy [67]. However, incorporating a high volume of filler can inevitably disrupt the polymer network backbone. This disruption can lead to the deterioration of the composite’s mechanical properties, including reduced flexibility, tensile strength, and toughness. Balancing the amount of filler to achieve the desired performance improvements while maintaining the integrity of the TPU matrix is a critical challenge in composite material design. Figure 5 presents the typical stress–strain curves, along with the tensile strength and elongation at break data. With just 0.5 wt% loadings of MXene, the tensile strength, yield strength, and hardness of TPU increased by 20%, 70%, and 10%, respectively, while maintaining its intrinsic elongation at break [68]. This indicates that a low dose of MXene can yield a homogeneous distribution. However, when higher loadings are required, further modifications are necessary to enhance compatibility with the TPU matrix.

### 4.3. Utilization of Modified MXene

Modified MXenes play a critical role in improving their compatibility with polymers such as the TPU matrix [69]. These modifications often include functionalizing MXene surfaces with organic molecules, inorganic materials, polymers, or other compounds to customize their properties. Functional groups on MXene surfaces can modify their chemistry, improving adhesion between MXene and the TPU matrix while maintaining desired functionalities such as electrical conductivity, mechanical strength, thermal stability, and flame retardancy.

#### 4.3.1. Functionalized MXene by Noncovalent Adhesion

**Figure 6:**
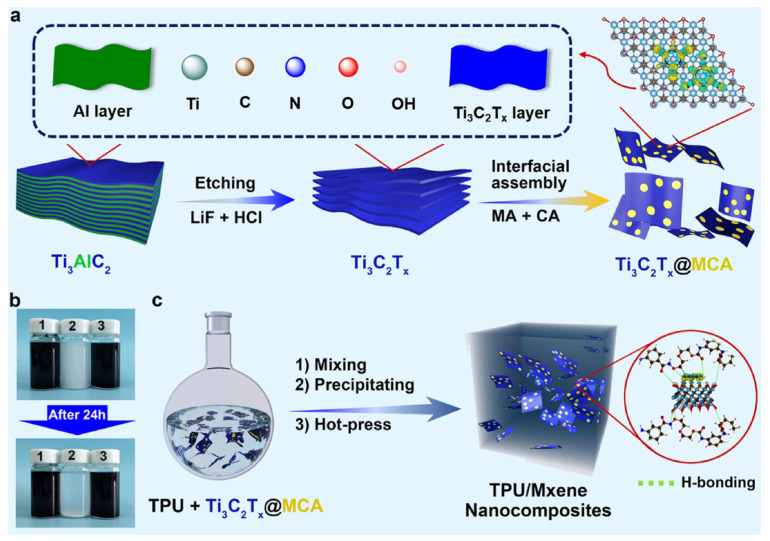
Schematic diagrams for (**a**) the synthesis of Ti_3_C_2_T_x_@MCA nanohybrid and (**c**) the preparation of TPU/Ti_3_C_2_T_x_@MCA nanocomposites; (**b**) digital photographs of (nano)additive dispersion: (b-1) Ti_3_C_2_T_x_ in DI, (b-2) MCA in DMSO and (b-3) Ti_3_C_2_T_x_@MCA in DMSO [74]. Copyright 2020. Reproduced with permission from Elsevier Science, Ltd.

Functionalized MXenes by hydrogen bonding involve altering the surface of MXene sheets following the selective removal of elements from MAX phases. This process reveals plentiful active terminal groups such as –OH, =O, and -F on MXene surfaces, which offer multiple sites capable of forming hydrogen bonds with other atoms or molecules [70,71,72,73]. These functional groups significantly enhance the compatibility of MXenes with TPU composites, including inorganic nanoparticles and organic materials, through strong and specific hydrogen bonding interactions. As depicted in Figure 6, Shi et al. [74] developed a multifunctional nanohybrid, Ti_3_C_2_T_x_@MCA, by engineering hydrogen bonding interactions between the hydroxyl (OH) groups on the MXene surface and the NH_2_/NH groups of melamine cyanurate (MCA). This nanohybrid was well dispersed within the TPU matrix. The formation of hydrogen bonds between Ti_3_C_2_T_x_@MCA and the TPU matrix enhances dispersion and fracture energy, leading to exceptional mechanical and fire-retardant properties.

**Figure 7:**
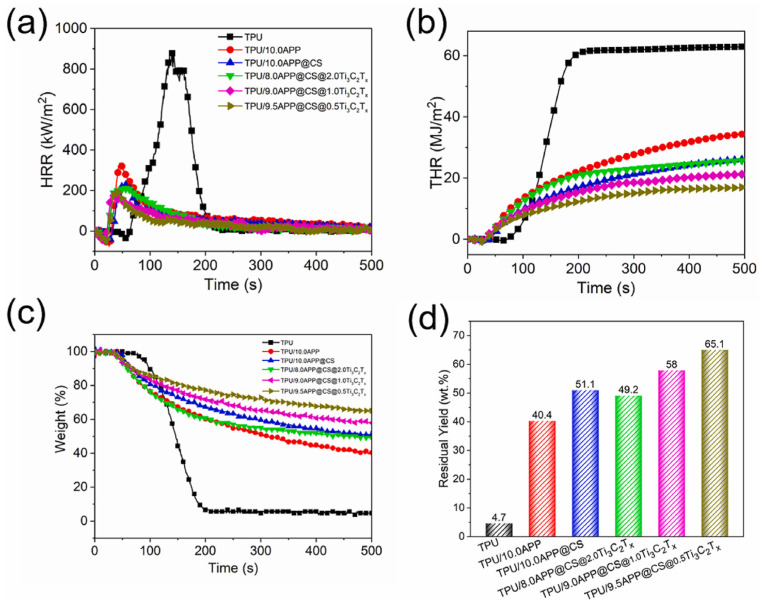
(**a**) HRR, (**b**) THR, (**c**,**d**) char residues of TPU and its composites [79]. Copyright 2021. Reproduced with permission from Elsevier Science, Ltd.

The improved dispersion of MXene is a crucial factor influencing the mechanical properties and flame-retardant efficiency of polyurethane nanocomposites [23,75,76,77]. As anticipated, Liu et al. [78] prepared Ti_3_C_2_T_x_-rGO hybrids via hydrogen bonding, significantly enhancing the thermal and flame-retardant performance of TPU nanocomposites. The resulting TPU/Ti_3_C_2_T_x_-rGO composites exhibited notable reductions in total smoke release (54.0%) and total carbon monoxide yield (46.2%), even with a low loading content of 2 wt%. Additionally, MXene can act as a “bridge” to link conventional flame retardants with TPU, thereby enhancing compatibility within the TPU matrix. Liu et al. [79] developed a core–shell-structured hybrid, referred to as APP@CS@Ti_3_C_2_T_x_, with the goal of enhancing interface compatibility within the TPU matrix. As shown in Figure 7, this hybrid imparts outstanding thermal stability and highly efficient fire retardancy to the TPU composite.

**Figure 8:**
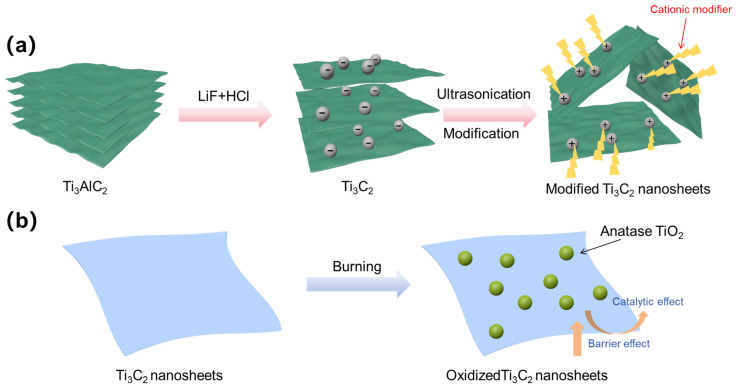
(**a**) Illustration for preparation of functionalized Ti_3_C_2_ (MXene) nanosheets with cationic agents. (**b**) Barrier effect of Ti_3_C_2_ nanosheets and the illustration of thermal oxidation of Ti_3_C_2_ nanosheets.

MXene surfaces become highly hydrophilic after acid etching, which poses a challenge as they are incompatible with most hydrophobic polymer materials. This inherent hydrophilicity often results in inadequate dispersion and bonding within polymer matrices, thereby limiting their effectiveness in composite materials. To address this issue, it is crucial to modify the surface properties of MXene nanosheets using suitable modifiers prior to compounding with polymers. Cationic reagents are commonly employed across various applications to improve the stability of micro- and nanofillers, control their shapes, and modify their surfaces by introducing diverse functional groups [80]. As depicted in Figure 8a, the negatively charged MXene layer can adsorb positively charged modifiers on its surface through electrostatic interactions, preventing layer stacking and thereby enhancing interfacial interactions with the TPU. Furthermore, Yu and colleagues [65] utilized two frequently employed cationic modifiers, cetyltrimethylammonium bromide (CTAB) and tetrabutylphosphonium chloride (TBPC), to modify MXene ultra-thin nanosheets. MXene can contribute to self-charring and react with the char from TPU, creating an effective barrier that significantly prevents polymer combustion and smoke release (Figure 8b). The enhanced flame-retardant properties and reduced smoke and toxicity hazards of these materials are attributed to their effective dispersion within the TPU matrix and the catalytic and “tortuous path” effects enabled by the modified MXene.

#### 4.3.2. Functionalized MXene by Covalent Bonding

**Figure 9:**
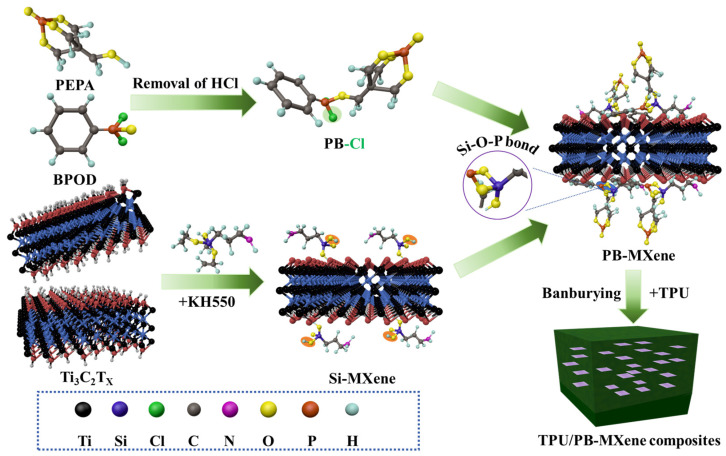
Illustration for the fabrication process of PB-MXene and TPU/PB-MXene composites.

In contrast to noncovalent bonding, covalent functionalization creates a more stable chemical structure in flame retardants, ensuring the performance of MXene-based polymer composites. This process involves chemically attaching molecules or functional groups directly to the MXene surface. The stability from strong chemical bonds between MXene nanosheets and functional groups makes them less likely to break or degrade under high temperatures and harsh conditions typically encountered during combustion. Covalent functionalization is essential for tailoring MXene to meet the diverse requirements of various technological applications effectively. As illustrated in Figure 9, Jiang et al. [81] developed a sandwich-structured PB-MXene by grafting organophosphorus (PB) onto the surface of MXene. The strong interactions between PB-MXene and the TPU matrix resulted in better dispersion and enhanced mechanical properties compared to the mixed and single systems of TPU/PB and TPU/MXene. Meanwhile, the peak heat release rate of the TPU/PB-MXene composite decreased by 72.6% compared to the pure TPU, indicating the presence of both condensed-phase and gas-phase flame-retardant mechanisms.

#### 4.3.3. Synergism between MXene and Flame-Retardant Compounds

The synergistic combination of MXenes with traditional flame retardants offers a multifaceted approach to improving the flame retardancy of polyurethane composites [82,83,84]. This strategy leverages the strengths of both MXenes and other flame retardants, resulting in materials that are more effective in preventing fire spread, reducing smoke production, and maintaining structural integrity under high temperatures. This phenomenon leverages the unique properties and mechanisms of each flame retardant to enhance the fire protection capabilities of the composite material in ways that would not be possible with any single component alone. Liu et al. [85] synthesized a phosphoramide-based compound (PPPA) through esterification and then prepared MXene-based hybrids. These hybrids demonstrated strong interface interactions within the TPU matrix, resulting in a notable enhancement in the thermal stability and flame retardancy of TPU composites. This improvement can be attributed to the thermal oxidation action and “tortuous path” effect of MXene, along with the quenching effect of PPPA. 

**Figure 10:**
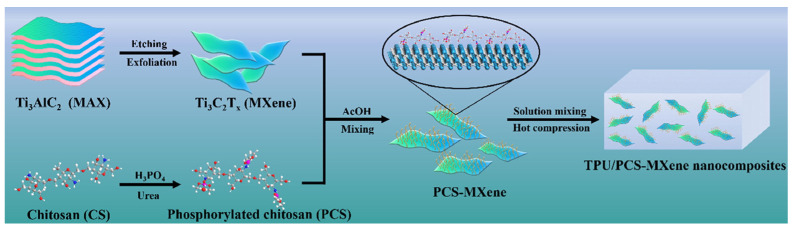
Illustration of the preparation of TPU/PCS-MXene nanocomposites [75]. Copyright 2022. Reproduced with permission from Elsevier Science Ltd.

Biomass materials such as phytic acid (PA) and chitosan (CH) are carbon-rich substances commonly employed as char-forming agents in flame-retardant systems [86,87,88]. These materials possess a high carbon content, which is crucial for their effectiveness in flame retardancy. During combustion, these substances undergo thermal decomposition, leaving behind a carbonaceous residue or char, which can also act as a physical barrier, hindering the transfer of heat and gases during combustion. Luo et al. [75] produced a phosphorylated bio-based chitosan functionalized MXene (Figure 10). The TPU/PCS-MXene composites demonstrated superior thermal stability and mechanical properties, alongside the effective suppression of smoke emission. The findings indicate that the flame retardancy of TPU composites was significantly enhanced through PCS modification, paving the way for the design of high-performance TPU materials. Therefore, the synergistic approach is promising for developing advanced materials that meet stringent fire safety standards and are suitable for various high-performance applications.

### 4.4. MXene-Based Flame-Retardant Polyurethane Foam Systems

MXenes, as a versatile 2D material, not only serve as an effective flame-retardant additive but can also be utilized as a coating material to enhance fire safety in polyurethane foam systems. When applied as a coating, MXenes form a protective layer on the surface of rigid polyurethane foam (RPUF) or other foam materials. This layer adheres strongly due to attractive forces, creating a barrier that significantly reduces flammability. The MXene coating acts by insulating the underlying material from heat and oxygen, thereby delaying ignition, slowing down flame spread, and reducing the release of smoke and toxic gases during combustion. The dual role of MXenes, functioning both as an integrated additive and an external coating, provides a comprehensive strategy for enhancing the fire safety of polyurethane foams, thereby broadening their use in high-risk environments. 

Wang et al. [22] fabricated MXene-coated flexible polyurethane foam (FPUF) by immersing the PUF into a suspension, followed by a drying process. The compact and uniformly distributed MXene nanosheets formed an effective flame-retardant coating on the PUF skeleton, significantly improving the anti-dripping performance of the coated PUF throughout the entire combustion process. However, due to the lack of interaction between adjacent layers, the coating content of the nanofillers is limited to a relatively low level. Lin et al. [89] incorporated oppositely charged chitosan to create an electrostatic attraction between the MXene layer and the chitosan layer, thereby strengthening the binding force between them. The resulting MXene/chitosan coating demonstrated excellent barrier and carbonization properties, effectively reducing smoke generation and significantly enhancing the fire safety of FPUF. 

**Figure 11:**
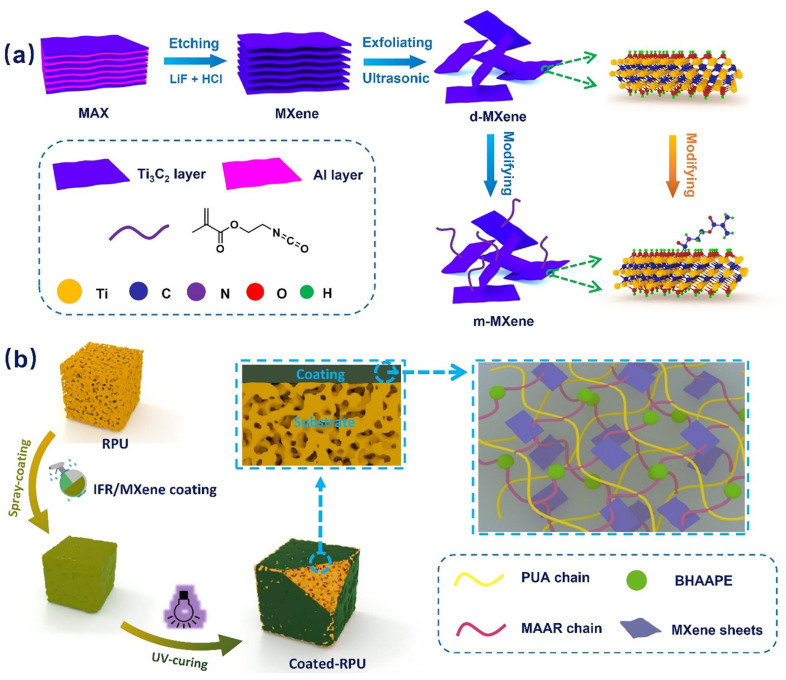
Schematic illustration of the fabrication procedure of (**a**) surface-modified MXene nanosheets and (**b**) IFR/MXene coated RPU foam [90]. Copyright 2020. Reproduced with permission from Elsevier Science, Ltd.

Additionally, spray coating technology has been utilized on rigid polyurethane foam (RPUF) foam to create a fire-retardant coating [90]. As illustrated in Figure 11, the coating slurry was prepared by blending UV-curable intumescent flame retardant with MXene and then spraying it onto the foam surface, forming a protective layer that effectively shields the internal matrix from heat exposure. The mechanical properties and dispersion are further improved due to the co-cross-linking networks between the IFR coating and MXene nanosheets.

### 4.5. Flame-Retardant Mechanism for MXene

The integration of MXenes into thermoplastic polyurethane (TPU) matrices represents a significant advancement in the development of flame-retardant materials. The influence of MXenes on TPU flammability is multifaceted, encompassing enhancements in thermal stability, modifications in combustion behavior, and alterations in smoke and gas emissions. The performance of MXenes as flame retardants in TPU is influenced by their loading levels and distribution within the polymer matrix. Optimal dispersion and concentration are key factors in maximizing the effectiveness of MXenes. Variations in these parameters can lead to differing outcomes in fire retardancy, making it crucial to establish the ideal conditions for incorporating MXenes into TPU.

MXene has emerged as an efficient flame retardant due to several key properties, including its excellent thermal stability, layered structure, superior catalytic activity, and tunable chemical properties. Research indicates that Ti-based substances derived from MXene, such as TiO_2_ particles formed during decomposition, show significant catalytic effects [78,91], effectively inhibiting the emission of toxic smoke during combustion. Furthermore, the “tortuous path” effect refers to the physical barrier created by MXene nanosheets within the polymer matrix. Additionally, MXene enhances the formation of stable char layers in the condensed phase, which acts as protective barriers, thereby improving the fire resistance of the material.

**Figure 12:**
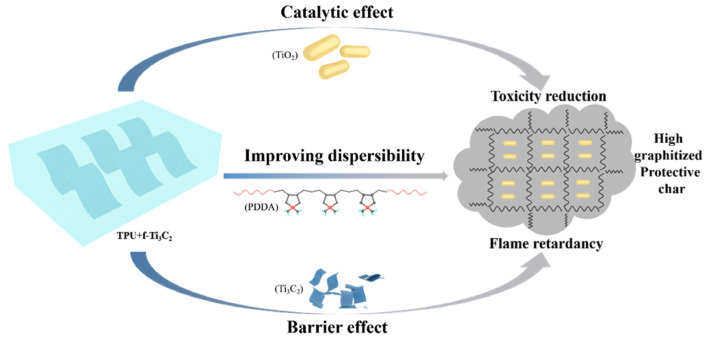
Scheme of proposed flame-retardant mechanism for f-Ti_3_C_2_ in TPU composites [92]. Copyright 2019. Reproduced with permission from Elsevier Science, Ltd.

Wang et al. [22] conducted a detailed comparative analysis of the elemental composition of pristine Ti_3_C_2_T_x_ film before and after undergoing combustion treatment to gain deeper insights into the flame-retardant mechanism of MXene in polymer composites. This study aimed to elucidate the changes and reactions occurring within the MXene structure when exposed to high temperatures during combustion. The results indicated that as the surrounding temperature increased, an oxidation reaction occurred and transformed Ti_3_C_2_T_x_ into TiO_2_. Similar results were observed in flame-retardant TPU/MXene composites. He et al. [92] incorporated Ti_3_C_2_T_x_ nanosheets into TPU composites, where the well-dispersed Ti_3_C_2_T_x_ acted as a physical barrier, increasing the distance and time required for heat, oxygen, and combustible gases to diffuse through the material (Figure 12). At elevated temperatures, Ti_3_C_2_T_x_ gradually transformed into TiO_2_ particles, which possess excellent catalytic properties and facilitate the conversion of lower-carbon pyrolysis products into highly graphitized chars.

Apart from fire hazards, the generation of various toxic smoke and harmful gases such as carbon monoxide (CO) and nitrogen oxides (NO_x_) from the degradation of TPU composites is also a significant concern. When subjected to heat and flames, MXenes promote the formation of a stable char layer on the surface of the material undergoing combustion. This char layer acts as a protective barrier that further inhibits the release of gases and reduces the intensity of the flame. It also contributes to reducing smoke production by trapping volatile components within the char matrix. Luo et al. [75] successfully synthesized PCS-modified MXene, which reduced CO production in TPU by 52.1%. Moreover, the high-aspect-ratio lamellar-modified MXene nanosheets inhibited the emission of volatiles and facilitated the formation of compact chars, thereby suppressing polymer degradation and smoke production. The mechanisms involved primarily include catalytic action, adsorption, and the “tortuous path” effect. 

## 5. Concluding Remarks and Future Aspects

The primary objective of this review is to investigate the progress in MXene flame retardants, focusing on their applications based on pristine MXene, functionalized MXene, and synergism with other flame-retardant compounds. MXene-based flame retardants have garnered significant attention due to their unique properties and effectiveness in enhancing flame retardancy in various polymer matrices. Pristine MXene has been shown to improve the flame-retardant properties of the TPU matrix due to its inherent thermal stability, layered structure, and catalytic activity. The incorporation of pristine MXene into the thermoplastic polyurethane (TPU) matrix has demonstrated a reduction in flammability, increased char formation, and decreased release of smoke and toxic gases during combustion. However, higher loadings require further modifications to enhance compatibility with the TPU matrix. Research efforts have focused on optimizing MXene dispersion, surface modification techniques (such as cationic modification and covalent bonding), and understanding the synergistic effects of TPU. These efforts have led to enhanced compatibility between MXenes and TPU, resulting in composites with improved performance and durability. Additionally, the integration of MXenes into a broader range of polymer matrices, such as epoxy resins, polyvinyl chloride (PVC), and polyethylene, could yield noteworthy improvements in fire resistance due to MXene’s inherent properties, such as high thermal stability, layered structure, and catalytic activity. These polymers, widely used in various industrial applications, present an opportunity for enhanced flame-retardant performance through innovative MXene-based formulations.

Despite the significant advances and growing attention MXene-based flame retardants have received in recent years, several issues remain unresolved.

(i). The large-scale production of MXene materials is still a challenge. The current synthesis methods, including the selective etching of MAX phases and subsequent delamination, are often expensive, time-consuming, and involve hazardous chemicals like hydrofluoric acid. These factors limit the commercial viability of MXene. Developing cost-effective, safe, and scalable production techniques is crucial for their widespread adoption in industry.
(ii). MXene materials can be prone to oxidation and degradation when exposed to air and moisture. This instability can compromise their flame-retardant properties over time, reducing their effectiveness in practical applications. Research is needed to improve the long-term stability of MXene, possibly through protective coatings or more stable surface modifications.
(iii). Functionalizing MXene surfaces to enhance their properties, such as compatibility with polymer matrices or improving flame retardancy, often involves complex chemical processes. These processes can lead to the introduction of impurities or defects, potentially having a negative impact on material performance. Developing simple, efficient, and reproducible functionalization methods is necessary to ensure consistent quality and functionality.
(iv). The potential environmental and health impacts of MXene production, use, and disposal are not fully understood. The synthesis process involves hazardous chemicals, raising concerns about safety and environmental contamination. Moreover, the long-term effects of MXene nanoparticles on human health and the environment need to be thoroughly investigated. Life cycle assessments and toxicological studies are necessary to evaluate the sustainability and safety of MXene-based flame retardants. Regulations and guidelines for the handling and disposal of MXene materials should be developed to mitigate potential risks.

To harness the exceptional performance of MXene in the TPU matrix, future efforts can focus on several key areas: (1) Cost-effective, scalable, and environmentally friendly synthesis methods for MXenes should be developed. Exploring alternative etching agents, green chemistry approaches, and continuous production processes could significantly enhance the commercial viability of MXene/TPU nanocomposites. (2) Developing strategies to protect MXenes from oxidation and degradation is crucial for improving their long-term stability. (3) Advancing interfacial engineering techniques to improve the dispersion and bonding of MXenes within the TPU matrix is critical. Using compatibilizers, surfactants, or coupling agents can enhance the interfacial interactions and ensure the uniform distribution of MXenes, thereby optimizing the mechanical strength and flame retardancy of TPU composites.

## Figures and Tables

**Table 1:** Comparison of material information and mechanical properties of various material systems.

Composition of Materials	Preparation Method	Fillers Content	Tensile Strength (MPa)	Properties	Ref.
GO/TPU	In situ polymerization	1 wt%	↑40.5%	Mechanical	[60]
CNT/TPU	Melt blending	1 wt%	↑20%	Mechanical + Thermal	[61]
TPU/MWCNTs TPU/MWCNTs@MXene	Pressing Foaming	10 wt%	TPU/MWCNTs@MXene > TPU/MWCNTs	Mechanical + conductivity	[62]
TPU/MXene@SnO_2_	Hot pressing	2%	↑26.8%	Mechanical + Flame Retardant	[63]
Ti_3_C_2_T_x_/TPU	Melt blending	0.5 wt%	↑41.2%	Mechanical + crystalline	[64]
Ti_3_C_2_T_x_/TPU	Melt blending	0.5 wt%	↑47.1%	Mechanical + Flame Retardant	[25]

## Data Availability

Not applicable.
